# Heterologous expression of pediocin/papA in *Bacillus subtilis*

**DOI:** 10.1186/s12934-022-01829-x

**Published:** 2022-05-28

**Authors:** Genyu Wang, Zhijun Guo, Xueqian Zhang, Hao Wu, XiuMei Bai, Hailiang Zhang, Richa Hu, Shaoliang Han, Yuanxiang Pang, Zi’ang Gao, Lili Yan, Cuiying Huang, Le Zhang, Chunli Pan, Xuelian Liu

**Affiliations:** 1 State Key Laboratory of Direct-Fed Microbial Engineering, Beijing, 100192 China; 2 Research Center of Feed Safety and Bioregulation Engineering Technology, Beijing, China; 3 Beijing Dabeinong Technology Group Co.Ltd., Beijing, 100192 China

**Keywords:** *Lactobacillus plantarum*, Pediocin, *papA*, *Bacillus subtilis*, Microbial cell factory, *Listeria monocytogenes*

## Abstract

*Listeria monocytogenes* is a food-borne pathogen. Pediocin is a group IIα bacteriocin with anti-listeria activity that is naturally produced by *Pediococcus acidilactic* and *Lactobacillus plantarum.* The *pedA*/*papA* gene encodes pediocin/plantaricin. In native hosts, the expression and secretion of active PedA/PapA protein rely on the accessory protein PedC/PapC and ABC transporter PedD/PapD on the same operon. The excretion machines were also necessary for pediocin protein expression in heterologous hosts of *E. coli*, *Lactobacillus lactis*, and *Corynebacterium glutamicum*. In this study, two vectors carrying the codon sequence of the mature PapA peptide were constructed, one with and one without a His tag. Both fragments were inserted into the plasmid pHT43 and transformed into *Bacillus subtilis* WB800N. The strains were induced with IPTG to secrete the fused proteins PA1 and PA2. Supernatants from both recombinant strains can inhibit *Listeria monocytogenes* ATCC54003 directly. The fused protein possesses inhibition activity as a whole dispense with removal of the leading peptide. This is the first report of active pediocin/PapA expression without the assistance of PedCD/PapCD in heterogeneous hosts. In addition, the PA1 protein can be purified by nickel-nitrilotriacetic acid (Ni-NTA) metal affinity chromatography.

**Supplementary Information:**

The online version contains supplementary material available at 10.1186/s12934-022-01829-x.

## Introduction

*Listeria monocytogenes* is a food-borne pathogen [1] that can grow at near-freezing temperatures [2]. *Listeria monocytogenes* contamination leads to listeriosis due to the inadequacy of conventional preservation methods. Bacteriocin-producing lactic acid bacteria [3] are considered promising for the control of *L. monocytogenes*. Bacteriocins are a subgroup of antimicrobial peptides that usually exhibit strong antimicrobial activity against microbes closely related to the producing bacteria [4]. Bacteriocins are classified into four groups according to their structure and modification. Nisin is a class Iα bacteriocin that undergoes posttranslational modification [5]. Pediocin (PA-1) is a class IIα bacteriocin, heat-stable, ribosomally synthesized, unmodified peptide [6], with anti-*Listeria* effects [7]. Nisin is a broad-spectrum bacteriocin and has shown efficacy in the control of clinically relevant methicillin-resistant *Staphylococcus aureus* (MRSA), vancomycin-intermediate *S. aureus* (VISA), and vancomycin-resistant Enterococcus (VRE) [3, 8]. Nisin is one of the most studied bacteriocins and the only bacteriocin approved for commercial use as a food additive [9–11]. Pediocin is a narrow-spectrum bacteriocin but is more potent than nisin against *L. monocytogenes* [12]. The pediocin-like group IIα bacteriocin gene is located in an operon of four genes that are necessary for bacteriocin production and secretion in native hosts [13]. The first gene is the structural prebacteriocin gene *pedA*/*papA* and is followed by the immunity gene *pedB*/*papB*, which encodes an immunity protein that protects the bacteriocin producer from its own bacteriocin [14]; the gene *pedC*/*papC*, which encodes an accessory protein with chaperone-like activity and ensures the formation of the correct disulfide bond in bacteriocin [15]; and the gene *pedD*/*papD*, which encodes an ABC transporter (ATP-binding cassette) necessary for the production and secretion of the active pediocin PA-1/PapA [16]. The whole prebacteriocin peptide PapA contains an N-terminal leading peptide and a C-terminal mature peptide. The mature peptide is site-specifically cleaved from the prepeptide and secreted into the medium by the ABC transport protein PedD. The mature peptide then displays bacteriocin activity. *Lactobacillus plantarum* Zhang-LL is a strain with anti-listeria activity isolated from fermented rice [17]. The gene responsible for this function was attributed to *papA* in megaplasmid pZL3. The structure and sequence of *papABCD* are nearly identical to those of *pedABCD* in *Pediococcus acidilactici, Pediococcus pentosaceus,* and *Pediococcus parvulus*. The *pedABCD/papABCD* operon is often located in the Mega-plasmid and undergoes horizontal transfer between strains[18] [19].

The pediocin gene has been introduced into *Lactococcus lactis* [20, 21], *E. coli* [22–24], and pediocin-negative *Pediococcus acidilactici* [24], where the ABC transporter protein encoding gene *pedD* [16] or lactococcin A secretion machinery were necessary for the activity of pediocin [21]. In *E. coli*, the mature peptide of pediocin Ach was fused in frame to the COOH terminus of the secretory protein maltose-binding protein (MBP) [22]. In *E. coli*, a periplasmic leaky *E. coli* strain had to be used to express and secrete the chimeric protein into the medium, which displayed listericidal activity. The *pedA* gene was also fused with the dihydrogolate reductase (*DHFR*) gene [25], the thioredoxin (*Trx*) gene [26, 27], and the green fluorescent protein (*GFP*) gene [28] on the 5´ side. The fused protein had no biological activity after IPTG induction. The fused protein was purified, and pediocin was then cleaved from the fused peptide. The pure pediocin peptide was demonstrated listericidal activity. Recently, Goldbeck et al. heterologously expressed the *pedA* gene in *Corynebacterium glutamicum*. The *pedACD* gene had to be inserted into the vector to ensure an active PedA peptide, which illustrated that PedCD peptides are also essential for the active PedA peptide in *C. glutamicum* [29]. The proteins PedC/PapC and PedD/PapD are 174 and 724 amino acids long, respectively, which are both larger than the PepA/PapA mature peptide (44 aa) itself. This is demanding both for vector construction and for the host. Therefore, it is necessary to establish a simple and rational expression system for *papA/pedA*. *B. subtilis* is a traditional fermentation host for food and industrial enzymes. *B. subtilis* strains have become attractive industrial organisms owing to their high growth rates, short fermentation cycle times, and ability to secrete protein into the extracellular medium [30, 31]. In addition, *B. subtilis* strains are generally regarded as safe (GRAS) because toxic byproducts such as lipopolysaccharides are not formed in their cells. The biochemistry, physiology, and genetics of *B. subtilis* are well known [32], which makes it a promising microbial cell factory for use with the developing genetic manipulation tools. WB800N is a *B. subtilis* strain with eight extracellular proteases (*nprE*, *nprB*, *aprE*, *epr*, *mpr*, *bpr*, *vpr*, *WprA*) knockout [33], which can secrete protein directly into the culture medium, no need to worry about degradation. Plasmid pHT43 (MoBiTech) has a signal sequence of the *amyQ* (α-amylase) gene, which leads the fused protein excreting out of cells. This system guarantee proteins are exported from the cytoplasm directly into the culture medium [34–36]. In the native host, the PA-1/PapA mature peptide was excreted into the extracellular medium. Therefore, the excretion ability might endow *B. subtilis* with a suitable host for PA-1/PapA. In this study, *papA*/*pedA* was introduced into *B. subtilis* by vector pHT43. The intact fused protein demonstrated anti-listeria activity directly without any further processing, as expected.

## Results

### Inhibitory effect of the supernatant of *L. plantarum *Zhang-LL on *L. monocytogenes*, *B. subtilis*, and *E. coli*

*Lactobacillus plantarum* Zhang-LL was isolated from fermented rice and showed a broad inhibitory effect on *L. monocytogenes*, *B. subtilis*, and *E. coli*. The supernatant of *L. plantarum* Zhang-LL was acidic at pH 4.4. It was neutralized to pH 7.0 using sodium hydroxide. The original and neutral supernatants of *L. plantarum* Zhang-LL were applied to test the inhibition effect. The original supernatant of *L. plantarum* Zhang-LL inhibited the growth of all three strains. However, only *L. monocytogenes* ATCC54003 was inhibited by the neutralized supernatant (Additional file 1: Fig S1A–C). Lactic acid is a natural product of *Lactobacillus* species [37]. This result demonstrated that the broad inhibition effect was from organic acids but not from pediocin/plantaricin. Inhibition of *L. plantarum* Zhang-LL bacteriocin was specific to *L. monocytogenes*. This result was consistent with a previous report of the specific anti-listeria activity of pediocin [24, 38]. The *papABCD* gene is located on megaplasmid pZL3 of *L. plantarum* Zhang-LL (GenBank: KJ767737.1). The structure and sequence of the *papABCD* gene are identical to those of the *pedABCD* gene in *Pediococcus acidilactici* and *Pediococcus pentosaceus* [19], as is the function of bacteriocin PapA. The active PapA mature peptide is released from inactive probacteriocin and secreted into the medium with the help of the accessory protein PedC/PapC and peptide cleavage/export ABC transporter PedD/PapD. The bacteriocin PapA does not inhibit *B. subtilis* WB800N, which makes it a suitable heterogeneous host for PapA expression.

### Introduction of the *papA *gene to *B. subtilis*

The mature PapA/PA-1 peptide contains 44 amino acids. The expression vector pHT43 encodes the signal peptide of α-amylase, which leads the fused protein out of the host cell [39]. Considering the activity of the fused protein, a His-tag plus a thrombin digestion site were inserted between the sequence of the signal peptide and *papA*. The fragment *papA1* was optimized according to codon usage preference in *B. subtilis*. The resulting plasmid pHT43-*papA1* was confirmed by sequence and transformed into competent *B. subtilis* WB800N using the Spizizen protocol [40]. Colony PCR was carried out to confirm the insertion of the *papA* gene into the recombinant strain. The positive clone of *B. subtilis* WB800N/pHT43-*papA1* was named *B. subtilis* DBN-SKL-PA1 and subjected to the induction of protein expression by IPTG. The fused protein, PA1, contains the signal peptide of α-amylase, a His-tag, a thrombin digestion site and the PapA mature peptide. PA1 was expected to be excreted from the host cell due to the signal peptide of α-amylase. Pure PapA mature peptide can be released from the fused protein when necessary. The supernatant was obtained by centrifugation and tested for listericidal activity.

The codon sequence of the PapA mature peptide was also amplified from the genome of *L. plantarum* Zhang-LL. The vector pHT43-*papA2* was constructed by inserting the codon sequence of the PapA mature peptide into pHT43. The plasmid pHT43-*papA2* was introduced into WB800N after sequence confirmation. A positive colony of *B. subtilis* WB800N/pHT43-*papA2* was confirmed by colony PCR and named *B. subtilis* DBN-SKL-PA2.

### Induction of papA expression in *B. subtilis*

**Fig. 1:**
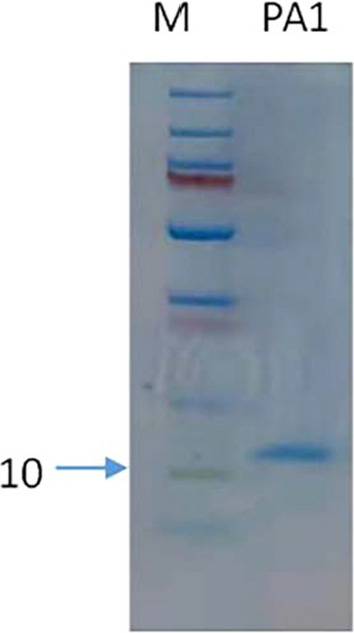
SDS-PAGE of PA1 peptide by Ni-NTA purification of supernatant of *B. subtilis* DBN-SKL-PA1

To induce gene *papA* expression in the recombinant strain, six concentrations of IPTG, 0.005, 0.01, 0.02, 0.05, 0.1, and 0.2 mM, were applied for both recombinant strains. The signal peptide of α-amylase on plasmid pHT43 is the secreting type, and the fused protein was produced by induction and transferred outside the host cell. The broth was collected at 2, 4, and 6 h after induction. The supernatant was obtained by centrifugation. *B. subtilis* DBN-SKL-PA1 produced a fused protein PA1, which consisted of a leading peptide of α-amylase, His tag, thrombin digestion site, and mature papA peptide. This protein was purified using Ni–NTA resin and SDS–PAGE (Fig. 1). Pure papA mature peptide can be obtained by the cleavage of thrombin on the NTA-Ni column. *B. subtilis* DBN-SKL-PA2 produces a protein PA2 consisting of a leading peptide of α-amylase and a mature peptide of papA.

### Inhibition of *L. monocytogenes*

**Fig. 2:**
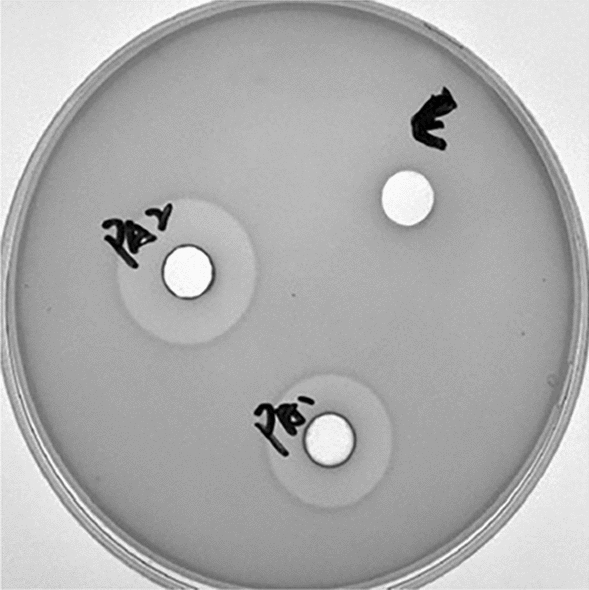
Inhibition of *L. monocytogenes* ATCC54003 by pediocin/plantarum using agar diffuse test. PA1, supernatant from induction culture of *B. subtilis* DBN-SKL-PA1 induced by 0.2 mM IPTG for 6 h; PA2, supernatant from induction culture of *B. subtilis* DBN-SKL-pA2 induced by 0.2 mM IPTG for 6 h; C, supernatant *B. subtilis* WB800N/pHT43 induced by 0.1 mM IPTG for 4 h

The *papA*/*pedA gene* is located in an operon harbouring four genes, *papABCD*/*pedABCD*. The *papCD*/*pedCD* genes are necessary for the expression of *papA*/*pedA*. It seemed that active PapA could not be produced in *E. coli* or *L. lactis* without a secretory apparatus [23]. In this study, strain DBN-SKL-PA1 and strain DBN-SKL-PA2 were induced with six concentrations of IPTG. One-millilitre samples were collected at 0, 2, 4, and 6 h. Supernatants of both strains were subjected directly to inhibition assay by *L. monocytogenes* ATCC54003. The results demonstrated that the two strains both produced active PapA-fused protein effectively. For both strains, the inhibition activity increased as the induction time prolonged. At 2 h, strain DBN-SKL-PA1 produced active protein only when the IPTG concentration was above 0.05 mM. At 4 h, 0.02 mM IPTG induced protein expression. At 6 h, active protein was induced even with 0.005 mM IPTG. The best inhibitory effect was obtained with 0.2 mM IPTG at 6 h (Additional file 1: Fig. S2A–C). Strain DBN-SKL-PA2 seemed to be more easily induced than DBN-SKL-PA1. The active protein PA2 was induced at 2, 4, and 6 h at all six concentrations. The best inhibition activity was similar for 0.05, 0.1 and 0.2 mM IPTG at both 4 and 6 h (Additional file 1: Fig. S2D, E). To compare the activity of the two fused proteins, the supernatant with 0.2 mM IPTG at 6 h was subjected to a gel diffusion assay. The result illustrated that PA2, produced by *B. subtilis* DBN-SKL-PA2, was stronger than PA1, produced by *B. subtilis* DBN-SKL-PA1, as shown clearly by the halo (Fig. 2). Most importantly, the results demonstrated that the fused PA1 and PA2 proteins expressed in *B. subtilis* were both active. Neither PapC/PedC nor PapD/PedD was necessary for active PapA/PA-1 in *B. subtilis*. The result also illustrated that the leading peptide did not have to be removed from the fused peptide to obtain a pure PapA peptide, which was quite different from the PapA-fused protein expressed in *E. coli*.

The fused protein PA1 contains a His tag, which was removed using a Ni-NTA column. Thrombin was also added to the Ni-NTA column to remove the leading peptide. The purified papA protein was eluted from the column. The eluate was subjected to agar diffusion and showed anti-listeria activity. Fused PA2 protein was not observed by SDS–PAGE (data not shown). Since both proteins can be used directly for anti-listeria, removing the leading peptide was not necessary to obtain an active protein. The DNA sequence was optimized for vector pHT43-*papA1* but not for pHT43-*papA2*. However, the inhibitory activity of the PA2-fused protein against *L. monocytogenes* was stronger than that of PA1, as shown by the inhibition zone (Fig. 2). DBN-SKL-PA2 was then used for further assays. IPTG (0.1 mM) was applied for batch fermentation.

### Batch fermentation of DBN-SKL-PA2 and inhibitory effect

**Fig. 3:**
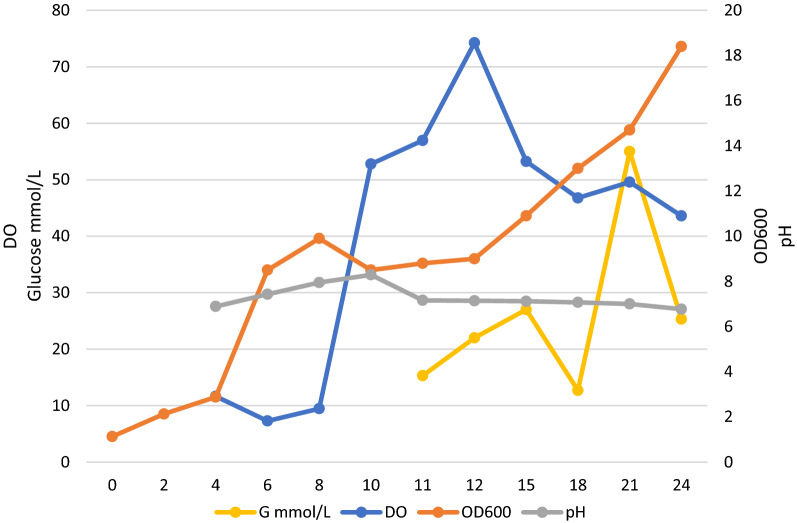
Substrate, dissolved oxygen, OD600, and pH during batch fermentation of DBN-SKL-PA2

Batch fermentation was performed with DBN-SKL-PA2 to demonstrate the feasibility of recombinant pediocin/PapA on a large scale. The initial culture conditions were similar to those of the shake flask. IPTG was added at 3 h to a final concentration of 0.1 mM. The culture was fed 100 g/L glucose and double the concentration of 2YT from 10 h, and IPTG was added at the beginning of the fed batch. A total of 1.6 L of fed medium was supplied. Phosphoric acid was added automatically to adjust the pH to below 7. Fermentation was carried out for 24 h. Dissolved oxygen, OD600, pH, and glucose concentration were recorded and are illustrated in Fig. 3.

The activity of PA2 fused protein was determined in culture supernatant at different time points by agar diffusion. The inhibition activity increased as the fermentation time increased in the gel diffusion assay (Additional file 1: Figs. S3A, B). *L. plantarum* Zhang-LL was fermented statically in MRS medium at 37 °C for 24 h, and the supernatant was used as a control. The inhibitory activity of the 21 h supernatant was stronger than that of the supernatant of *L. plantarum* Zhang-LL.

To assay the dry mass content, 10 g (9.05 mL) of supernatant was subjected to moisture measurement. Moisture was 96.80% for the supernatant of the 24 h fermentation broth, which resulted in 0.32 g of dry matter. Therefore, the total dry mass content in the original supernatant was 0.035 g/mL. The remaining powder was redissolved in sterilized PBS to a final concentration of 0.1 g/mL, i.e., in 3.2 mL of PBS, which was 2.83 times the concentration of the original supernatant. The drying duration was nearly 40 min at 105 °C. To investigate whether the activity was destroyed by heating to dryness, the rehydrated solution was subjected to gel diffusion. The results demonstrated that the activity was maintained after heating to dryness at 105 °C. Moreover, the rehydrated solution showed the largest halo (Additional file 1: Fig. S3B).

**Fig. 4:**
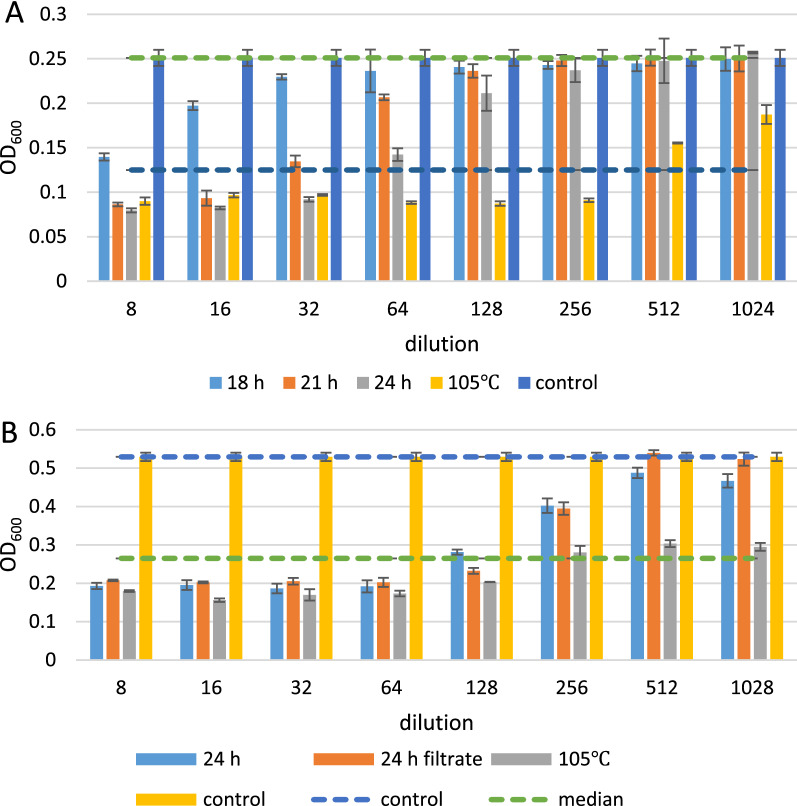
Growth inhibition of *L. monocytogenesis* ATCC54003 by twofold dilution supernatant of DBN-SKL-PA2 from batch fermentation. Value of OD_600_ of the indicator strain was measured by microplate reader in triplicate. 18 h, 21 h, and 24 h, were supernatant collected at 18, 21, and 24 h. 105 °C was supernatant of 24 dried under 105 °C, and rehydrated in sterilized PBS. 24 filtrate was 24 h supernatant sterilized by Millipore filter (0.22 μm).** A** *L. monocytogenesis* ATCC54003 was incubated with the supernatant of 18 h, 21 h, 24 h for 6 h.** B** *L. monocytogenesis* ATCC54003 was incubated with the supernatant of 24 h, 24 h filtrate, and 105 °C for 21 h

To determine the minimal inhibitory concentration (MIC) of the supernatant, the supernatant collected at 18, 21, and 24 h and the rehydrated solution were subjected to a standard microtiter plate assay. The cells were grown at 37 °C for 6 h. The result confirmed that inhibition activity improved with prolonging fermentation time (Fig. 4A). The MIC was a 64-fold dilution of the original supernatant of the 24 h fermentation broth. The original dry mass content was 0.035 g/mL, which implied that the MIC was 547 μg/mL total dry mass for the final fermentation broth. For rehydration solution, the MIC was between 256- and 512-fold dilutions, which equalled 195–390 μg/mL total mass. Therefore, the rehydrated solution of PA2 demonstrated the best activity. The inhibitory activity of rehydrated PA2 was nearly 1.4–2.8 times that of the original PA2. Pediocin/PapA was stable after heating at 80 °C for 1 h and at 100 °C for 10 min [13] [41]. The results here illustrated that the activity of PA2 can be retained at 105 °C for 40 min, which is more tolerance than previous reports.

The 24 h filtrate was obtained by sterilization of the 24 h supernatant using a 0.22 μm filter from Millipore. The 24 h supernatant, 24 h filtrate, and 105 °C rehydrated solution were subjected to a microtiter plate assay. The microtiter plate was incubated at 37 °C for 21 h. The inhibitory activity of the 24 h filtrate was similar to that of the 24 h supernatant. The inhibitory activity became more pronounced as the incubation period was prolonged. *L. monocytogenes* was inhibited by 128-fold dilution both for 24 h and 24 h filtrate, which equalled 273 μg/mL total dry mass for 24 h fermentation broth. For the 105 °C rehydrated solution, it was difficult to determine the MIC at 21 h. A 1028-fold dilution was shown to be effective with a little less than 50% inhibitory activity, which equalled 97.3 μg/mL rehydrated solution (Fig. 4B). The 24 h filtrate was further used for scanning electronic microscopy.

### Scan electronic microscopy of *L. monocytogenes* ATCC54003

**Fig. 5:**
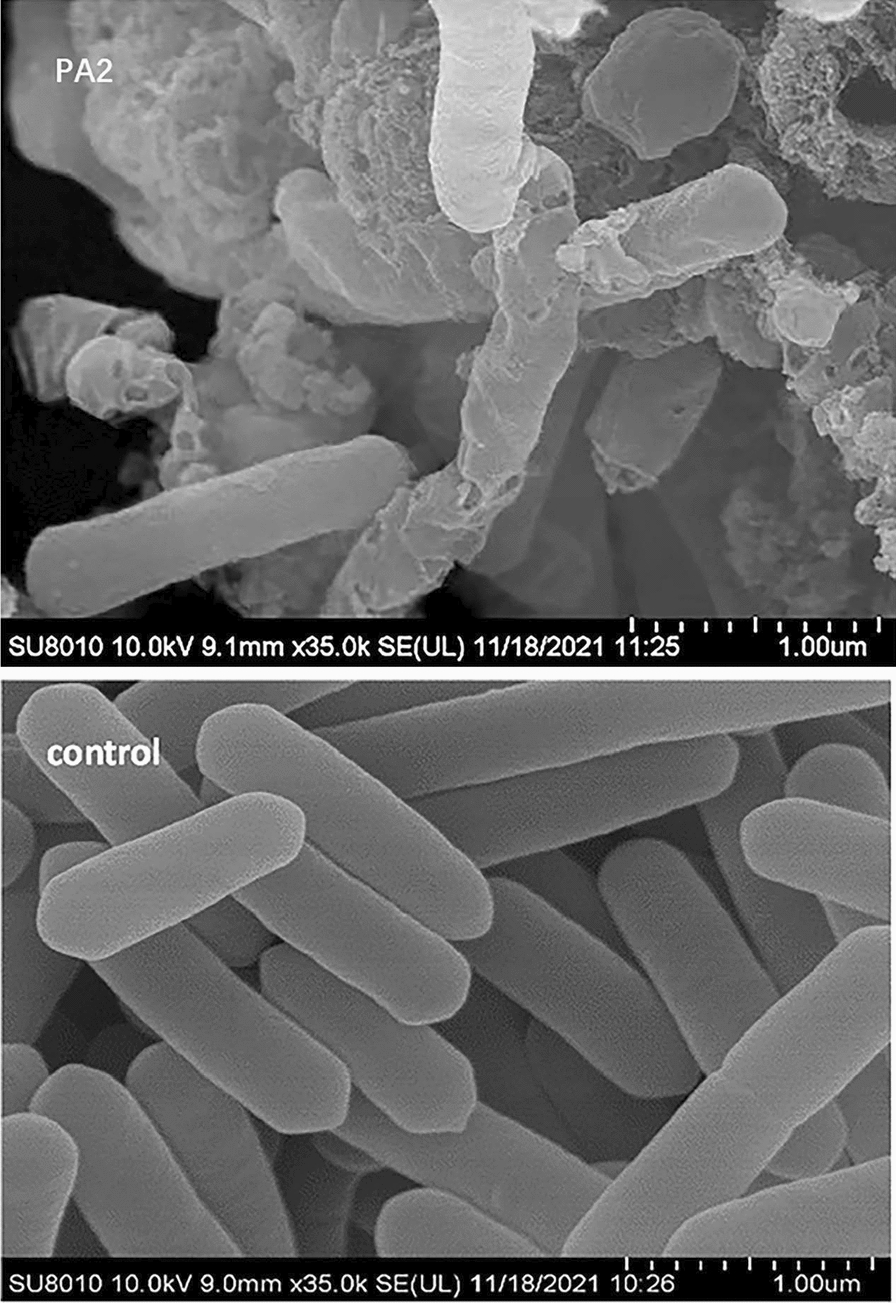
Scan electron microscope of *L. monocytogenes* ATCC54003. A, *L. monocytogenes* ATCC54003 cells incubated with supernatant of recombination protein PA2. B, control, *L. monocytogenes* ATCC54003 cells

*Listeria monocytogenes* ATCC54003 was grown to the exponential phase. The supernatant of the 24 h fermentation broth was sterilized by filtering to remove possible *B. subtilis* DBN-SKL-PA2 cells. The 24 h filtrate of PA2 was added to the culture as described in the microtiter plate assay and incubated for another 3 h. Cells were collected and subjected to SEM. The untreated cells were intact and smooth under SEM (Fig. 5). In contrast, cells were underwent severe damage when coincubated with PA2. The biofilm was disrupted, and the cell collapsed due to the outflow of cytoplasm (Fig. 5). The apparently unbroken cells were crude and elongated. This implied abnormal cell division. Cell division can be observed in the control cells, while no septation was observed in PA2 treated cells. The cell became long, and the length was more than 5.69 μm, which was more than twice that of the control (2.46 μm) (Additional file 1: Fig. S4). The destination of these cells should be broken due to a disordered cell cycle. Class IIa bacteriocins have been reported to act on the cytoplasmic membrane of gram-positive cells, dissipating the transmembrane electrical potential by forming pores [42]. The profile of *L. monocytogenes* ATCC54003 under PA2 in this study was consistent with previous reports.

## Discussion

In this study, the codon sequence of the PapA/pediocin mature peptide was introduced into *B. subtilis* WB800N. After induction, the supernatant was obtained and showed anti-listeria activity. This was an advance because it was the first time an actively secreting pediocin peptide was used in a heterogenous host without cotransformation of other secreting machinery. Pediocin is a secretion-type peptide in native hosts. The *pedA*/*papA* gene is naturally located in an operon that consists of three additional genes, *pedBCD*/*papBCD*. In native hosts, PapD protein is responsible for the cleavage of the leading PapA peptide and the excretion of the mature PapA peptide [16]. The excretion system was also indispensable for active PapA protein in *L. lactis* [20], *E. coli* [23]*,* and *C. glutamicum* [29]. The total amount of the PedC/PapC and PedC/PapC proteins was 20 times larger than that of the PedA/PapA mature peptide, which implied that a 20-fold redundant sequence had to be introduced for the active pediocin PedA/PapA. This is a burden both for vector and heterogenous expression hosts. In this study, an *E. coli*-*B. subtilis* shuttle vector pHT43 was applied. Vector pHT43 harbours a signal peptide of α-amylase. The signal peptide was secreted out of cells, as described in previous report [34], together with the peptide. The fused peptide retained its activity. This study demonstrated that *B. subtilis* is a superior host for protein secretion.

In *E. coli*, PapA was expressed as a fused peptide. The fused proteins were inactive, and it was necessary to purify and cleave the fused proteins to obtain pure PapA mature peptide. Then, the pure PapA peptide can inhibit *L. monocytogenes* [25, 27, 28]. The class IIα bacteriocins are produced as prebacteriocin with an N-terminal extension, which is removed by site-specific proteolytic cleavage during export. The mature bacteriocin is then secreted [6]. In this study, the secreting vector pHT43 was used to transform *B. subtilis*. The supernatant possessed anti-listeria activity after induction. We did not express the PapA mature peptide intracellularly in *B. subtilis*. Since the natural mature peptide is secreted, its activity might be maintained in the secreted form. Otherwise, the activity may also be attributed to the host *B. subtilis*. It is unclear if activity can be maintained in a cytoplasmically expressed PapA/pediocin protein in *B. subtilis*, which should be explored in future research.

A His tag and a thrombin digestion site were inserted between the signal peptide and the mature PapA peptide at first, in case the signal peptide had to be removed to release the active PapA. The fused peptide PA1 can effectively inhibit *L. monocytogenes* directly, which implied that the signal peptide did not hamper the function of PapA. Then, a “simple” plasmid was constructed, in which only the pure codon sequence of the PapA mature peptide was inserted into pHT43 to obtain pHT43-*papA2*. The fused protein PA2 contained a signal peptide of α-amylase and mature PapA peptide. To our surprise, the inhibitory activity of PA2 was superior to that of PA1, although the DNA sequence for pHT43-*papA1* was optimized according to the codon usage preference of *B. subtilis*. Strain DBN-SKL-PA2 was easily induced by IPTG at various concentrations. The supernatant possessed anti-listeria activity. This result implied that the difference in codon use frequency did not influence the expression level in *B. subtilis*, at least for the *papA* sequence. On the other hand, it has yet to be explored whether the His fragment influences anti-listeria activity. In addition, the activity may reflect the level of protein expression. The activity of PA2 was stronger, and the supernatant could be used directly because of the “GRAS” property of *B. subtilis*. PA2 was employed for the latter assay in this study. The induction condition of PA1 was not exploited fully. The MIC was assayed according to the total dry mass of the *B. subtilis* DBN-SKL-PA2 supernatant. Nevertheless, pure PapA mature peptide can be obtained from the PA1 fused peptide by His-Ni affinity chromatography and thrombin digestion. The induction conditions should be optimized further for *B. subtilis* DBN-DKL-PA1 if pure PapA/pediocin protein is desired.

*B. subtilis* is generally regarded as safe. Therefore, in this study, the whole supernatant was used to measure the inhibitory effect. The supernatant was placed at 105 °C for 40 min to measure the moisture content. We found that pediocin is thermotolerant. In a previous study, pediocin/PapA was stable during storage in a refrigerat, or by heating at 80 °C for 1 h and at 100 °C for 10 min [13] [41]. Therefore, the rehydrated solution was subjected to an inhibition assay. The result illustrated that activity was retained in the heated powder, which can inhibit *L. monocytogenes* after rehydration. This result implied that the PapA protein was thermotolerant, similar to carotene. Furthermore, the activity seemed to be boosted. The minimal inhibition concentration was lower for the rehydrated solution than for the original supernatant. There are four cysteines in mature papA/pediocin peptides. These cysteines form two disulfide bonds, which endow PapA/pediocin with considerable stability [13]. *L. monocytogenes* was not inhibited by the supernatant of *B. subtilis* WB800N/pHT43 in the gel diffusion assay (Additional file 1: Fig. S2). Therefore, the inhibitory activity of the rehydrated solution was still attributed to the PapA-fused protein. However, it is unclear why the activity was improved after heating to dryness. Further study should explore what happens to the PapA peptide under heating. Perhaps some toxic compound was produced during heating. Pediocin has been studied as a natural antimicrobial for food protection [38, 43]. Thermostability is an advantage for food processing and for concentrating the fermentation supernatant. However, a cell toxicology assay must be performed before pediocin can be used as a preservative.

## Conclusion

Two recombinant strains, *B. subtilis* DBN-SKL-PA1 and *B. subtilis* DBN-SKL-PA2, were constructed to express pediocin/PapA in *B. subtilis*. The strains can be induced to excrete PapA fused protein to the medium. The supernatant of the medium possessed anti-listeria activity. The fused pediocin/PapA fused protein was thermotolerant.

## Materials and methods

### Bacterial strains, plasmids, and primers

The bacterial strains, plasmids, and oligonucleotides used in this study are listed in Table 1. *L. plantarum* Zhang-LL was grown at 37 °C under static conditions for 24 h in MRS broth (tryptone 10 g, beef extract powder 10 g, yeast extract 5 g, glucose 20 g, sodium acetate 5 g, diamine citrate 2 g, Tween-80 1 g, K_2_HPO_4_ 0.4 g, MgSO_4_ 0.58 g, MnSO_4_ 0.29 g, and CaCO_3_ 20 g. The components were dissolved in ddH_2_O and adjusted to pH 6.3, and ddH_2_O was added to a final volume of 1 L). *L. monocytogenes* ATCC54003 was grown at 37 °C for 16 to 24 h in TSBYE medium (Hopebio, China). *B. subtilis* WB800N was grown at 37 °C in 2YT medium (tryptone 16 g, yeast extract 10 g, NaCl 5 g; the components were dissolved in ddH_2_O, adjusted to pH 6.3, and ddH_2_O was supplied to a final volume of 1 L). The upper three strains and vector pHT43 were generous gifts of Dr. Yuanhong Xie at Beijing University of Agriculture. *E. coli* Trans10 (TransGen Biotech, Beijing) was grown at 37 °C in LB medium for the appropriate time. Ampicillin and chloramphenicol were added at 100 μg/mL and 5 μg/mL, respectively, when necessary.

**Table 1:** Strains, plasmid, and primers used in this study

Strain and plasmid	Description	Source
Strain		
*E. coli* Trans10		Transgen
*L. plantarum* Zhang-LL		Jin et al. [17]
*B. sbutilis* WB800N	*nprE aprE epr bpr mpr::ble nprB::bsr Δvpr wprA::hyg cm::neo; NeoR*	Dr. Xie
*B. sbutilis* DBN-SKL-PA1	*B. sbutilis* WB800N with plasmid pHT43-papA1	This study
*B. sbutilis* DBN-SKL-PA2	*B. sbutilis* WB800N with plasmid pHT43-papA2	This study
*L. monocytogenes* ATCC 54003		Dr. Xie
Plasmid		
pHT43	Cm for *B.subtilis*, Amp for *E.coli*, P*grac01* promoter, modified signal peptide of α-amylase	Dr Xie
pHT43-papA1	pHT43 with His + Xa site + papA mature peptide cds	This study
pHT43-papA2	pHT43 with papA mature peptide cds	This study
Primer		
PAF	5′-CGGGATCCAAATACTACGGTAATGGGGT-3′	
PAR	5′-GCTCTAGATTGTTTAATATGTTCCGACT-3′	
P43F	5′-CGGACAGTTTCGTTCAGA-3′	
P43R	5′-TGAGTATCTTCTTCCGTGAT-3′	

### Plasmid construction

Two plasmids were constructed to express the PA-1/PapA mature peptide in *B. subtilis* WB800N. *E. coli*-*B. subtilis* shuttle vector pHT43 was used for vector construction and expression. The fragment of *papA1* consisted of a codon sequence from the PapA mature peptide and a His tag plus a thrombin reorganization site at the 5′´ side. The whole fragment *papA1* was optimized according to codon usage frequency and synthesized in Genewiz (China), which was then inserted into vector pHT43 between the *Bam*H I and *Xba* I sites. The vector pHT43-*papA1* was verified by sequencing with primers p43F and p43R. In another plasmid, pHT43-*papA2*, the sequence encoding the PapA mature peptide was amplified with the primers PAF and PAR from the genome of *L. plantarum* Zhang-LL. The PCR product was digested with *Bam*HI and *Xba*I and ligated into pHT43 digested with the same enzymes. The *papA* gene was used according to KJ767737/AII26511.1. The sequences of *papA1* and *papA2* are listed in Additional file 1: Table S1.

### Preparation of competent *B. subtilis* WB800N and transformation

*B. subtilis* WB800N was transformed according to the protocol of Spizizen [44]. In brief, one colony of *B. subtilis* WB800N was picked from a petri dish and inoculated in growth medium I (GM I, 1 × minimal mineral salt (g/L, K_2_HPO_4_, 14; KH_2_PO_4_, 6, (NH_4_)_2_SO_4_, 2; trisodium citrate·2H_2_O, 1; MgSO_4_·7H_2_O, 0.2) 95 mL, 50% glucose 1 mL, 5% acid hydrolysed casein 0.08 mL, 10% yeast juice 1 mL, L-Trp (2 mg/mL) 2.5 mL). Cells were cultured at 30 °C and 100 rpm for 16 h. Two millilitres of o/n GM I culture was inoculated into 18 mL of fresh GM I (10%) and incubated at 37 °C and 200 rpm for 3 h. Ten millilitres of the abovementioned culture was inoculated into 90 mL of GM II (1 × minimal mineral salt 97.5 mL; 50% glucose, 1 mL; 5% acid hydrolysed casein, 0.08 mL; 10% yeast juice, 0.04 mL; 2 mg/mL L-Trp, 0.5 mL; 500 mmol/L MgCl_2_, 0.5 mL; 100 mmol/L CaCl_2_, 0.5 mL) and grown at 37 °C and 100 rpm for 90 min. Bacteria were collected by centrifugation at 5000×*g* for 10 min. The sediment was suspended in 10 mL of GM II, which were competent WB800N cells. Then, 0.5 ~ 1 μg of plasmid was added to 500 μL of competent cells and mixed thoroughly. The system was incubated at 37 °C and 80 rpm for 30 min. The culture was spread on LB/CM agar and incubated at 37 °C for 12–16 h until colonies appeared. Positive transformants were confirmed by colony PCR.

### Inducible expression and purification of papA protein

A single colony of the positive recombination strain was inoculated into 2TY/CM liquid medium and cultured at 37 °C and 200 rpm overnight. The broth was inoculated into fresh 2TY/CM liquid medium and cultured for another 3 h. IPTG was added to the broth to induce protein expression at 30 °C and 200 rpm to final concentrations of 0.005, 0.01, 0.02, 0.05, 0.1, and 0.2 mM. Samples were withdrawn at 0, 2, 4, and 6 h. Samples were centrifuged at 12,000 rpm for 5 min and stored at 4 °C until further assay. Fused protein with a His tag was purified using Ni–NTA Resin (Thermo Scientific) according to the user’s guide.

### Antimicrobial activity assay

The bacteriocin activity of the induced supernatant was assessed by agar diffuse and standard microtiter plate assays. *L. monocytogenes* ATCC 54003 was grown in TSB-YE medium overnight at 37 °C and diluted 1:25 in fresh TSB-YE prior to the assay. When the optical density at OD_600_ was 0.05 ± 0.01, a tenfold dilution series of culture was performed. A 100 μL dilution was spread on a TSB-YE agar plate and grown overnight. Mueller–Hinton (MH) soft agar was added to an appropriate volume of *L. monocytogenes* ATCC 54003 solution to a cell concentration of 10^5^ CFU/mL. The medium was plated on a petri dish with an Oxford cup set beforehand. Then, 100 μL supernatant of expressed protein was added to the wells for agar diffusion. The petri dishes were incubated at 37 °C for 16–20 h, and antibacterial activity was observed as a halo of inhibition in the bacterial lawn formed around the sample. For the microtiter plate assay, a twofold dilution series of samples (100 μL) was mixed with 100 μl of the indicator strain (10^5^ CFU/mL) in sterilized 96-well plates. The plates were incubated at 37 °C for 5–6 h or 21 h. Growth was monitored by measuring the OD_600_ using an Epoch plate reader (BioTek). Bacteriocin activity in supernatants was determined in a semiquantitative manner according to 50% inhibition of the indicator strain [45].

### Cultivation in bioreactors

Fed-batch cultivation was carried out in a 30 L bioreactor (Baoxin, Shanghai) stirred tank bioreactor with an initial working volume of 15 L. A single colony was grown in 2YT medium supplemented with 5 μg/mL chloramphenicol in Erlenmeyer flasks with aeration at 37 °C and 200 rpm overnight to obtain a primary seed culture, which was then inoculated into 300 mL of 2YT/CM liquid medium and grown for 8 h. The preculture was inoculated with the 15 L 2YT/CM batch culture in a 30 L bioreactor and stirred at 200 rpm and 37 °C. IPTG was added after 3 h to a final concentration of 0.1 mM, and the temperature was lowered and kept at 30 °C. Then, 20% phosphoric acid was added automatically to adjust the pH to below 7. Dissolved oxygen was controlled by adjusting the stirrer speed and aeration rate from 200 ~ 300 rpm. The culture was fed 100 g/L glucose and double the concentration of 2YT from 10 h, and IPTG was added at the beginning of the fed batch to a final concentration of 0.1 mM. A total of 1.6 L of medium was fed. Dissolved oxygen and pH were recorded from the control panel of the bioreactor. The OD and glucose concentration were assayed. The sample was centrifuged, and the supernatant was stored at 4 °C for strain inhibition. Fermentation was conducted for 24 h. The broth was collected, and the supernatant was stored at 4 °C. To assay the dry material content in final broth, 10 g of supernatant of the 24 h fermentation broth was subjected to moisture measurement on a Moisture Analyzer (METTLER TOLEDO, HE53). The remaining powder was redissolved in sterilized PBS (Thermo Fisher).

### Scan electronic microscope

*Listeria monocytogenes* ATCC 54003 was grown in TSB-YE medium at 37 °C and 180 rpm overnight. One millilitre of culture was inoculated into 50 mL of fresh TSB-YE and grown to an OD_600_ of 0.6. The supernatant of the PapA-fused protein was sterilized by filtering and added to broth. The mixture was incubated at 37 °C for 3 h. A culture of *L. monocytogenes* ATCC 54003 without PapA protein was grown simultaneously as a control. Cells were harvested and washed with PBD, fixed with 2.5% (V/V) glutaraldehyde, and incubated at 4 °C o/n. After dehydration by gradient alcohol solutions, the samples were freeze-dried and coated with gold. The specimens were then examined using a scanning electronic microscope (SV8010, HITACHI).

### SDS–PAGE and Coomassie brilliant blue staining

SDS–PAGE was performed with Tris-Tricine 4–20% (Thermo Scientific) according to the manufacturer’s protocol. Samples were mixed with sample buffer and heated to 70 °C for 10 min before loading onto the gels. After electrophoresis, the gels were soaked in fast staining solution (Tiangen, Beijing), boiled for 1 min, and incubated at room temperature for 30 min. The gels were then washed with ddH_2_O, boiled for 1 min, and incubated at room temperature for 30 min. The washing step was repeated until clear bands were observed.

### Strain accession number

Strain *B. subtilis* DBN-SKL-PA1 and *B. subtilis* DBN-SKL-PA2 were deposited in the China General Microbiological Culture Collection Center (CGMCC) under CGMCC No. 23101 and CGMCC No. 23102.

## Supplementary Information

**Additional file 1.** **Table S1**: Gene papA sequences used in this study; **Figure S1**: Inhibition of supernatant of *Lactobacillus plantarum* Zhang-LL on *Bacillus subtilis*WB800N, *E.coli* K99, and *Listeria monocytogenes* ATCC54003; **Figure S2**: Inhibition of supernatant of various concentration of IPTG and induction time on *L. monocytogenes* ATCC54003; **Figure S3**: Gel diffusion assay of batch fermentation supernatant on *L. monocytogenes*ATCC54003. **Figure S4**: Scan electron microscope of **L. monocytogenes** ATCC54003.
