# Supplementary material for: Heterologous expression of pediocin/papA in Bacillus subtilis
Source: Microb Cell Fact. 2022 May 28;21:104. doi: 10.1186/s12934-022-01829-x (PMC9148482; doi:10.1186/s12934-022-01829-x)
Supplement: Supplementary file 1 — Additional file 1. Table S1: Gene papA sequences used in this study; Figure S1: Inhibition of supernatant of Lactobacillus plantarum Zhang-LL on Bacillus subtilisWB800N, E.coli K99, and Listeria monocytogenes ATCC54003; Figure S2: Inhibition of supernatant of various concentration of IPTG and induction time on L. monocytogenes ATCC54003; Figure S3: Gel diffusion assay of batch fermentation supernatant on L. monocytogenesATCC54003. Figure S4: Scan electron microscope of L. monocytogenes ATCC54003. [file 12934_2022_1829_MOESM1_ESM.docx]

Supplementary data to

**Heterologous expression of pediocin/papA in *Bacillus subtilis***

Genyu Wang^1,2,3^*, Zhijun Guo^1,2,3^, Xueqian Zhang^1,2,3^, Hao Wu^1,2,3^, XiuMei Bai^1,2,3^, Hailiang Zhang^1,2,3^, Richa Hu^1,2,3^, Shaoliang Han^1,2,3^,Yuanxiang Pang^1,2,3^, Zi’ang Gao^1,2,3^, Lili Yan^1,2,3^, Cuiying Huang^1,2,3^, Le Zhang^1,2,3^, Chunli Pan^1,2,3^, Xuelian Liu^1,2,3^*

1, State Key Laboratory of Direct-Fed Microbial Engineering, Beijing, China 100192

2, Research center of feed safety and bio-regulation engineering technology, Beijing

3, Beijing Dabeinong Technology Group Co.Ltd.,Beijing 100192

*: Corresponding author

Email address: [wgymap@126.com](mailto:wgymap@126.com); liuxuelian@dbn.com.cn

**Supplementary Data**

**Table S1**: **Gene papA sequences used in this study**

| seq | Sequence (5´→3´) | description | Size [bp] |
| --- | --- | --- | --- |
| papA1 | CATCACCATCACCATCATTCAAGCGGCCTGGTTCCGAGAGGCTCAAAATATTATGGCAATGGCGTTACATGCGGCAAACATTCATGCTCAGTTGATTGGGGCAAAGCAACAACATGCATTATTAATAATGGCGCAATGGCATGGGCAACGGGCGGCCATCAAGGCAATCATAAATGCTAA | optimazied sequence of His tag + thrombin + Codon papA mature peptide | 192 |
| papA2 | AAATACTACGGTAATGGGGTTACTTGTGGCAAACATTCCTGCTCTGTTGACTGGGGTAAGGCTACCACTTGCATAATCAATAATGGAGCTATGGCATGGGCTACTGGTGGACATCAAGGTAATCATAAATGCTAG | Codon sequence of papA mature peptide | 135 |


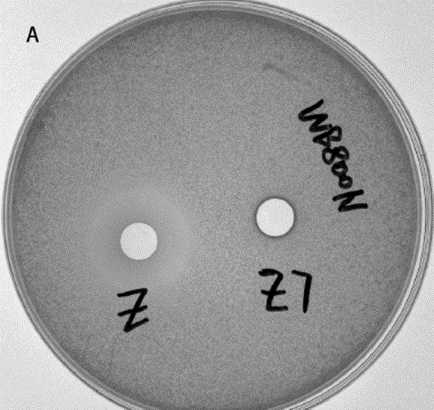

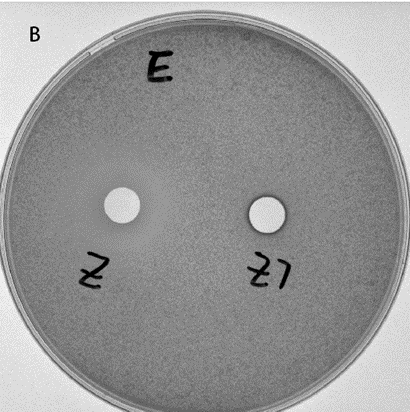


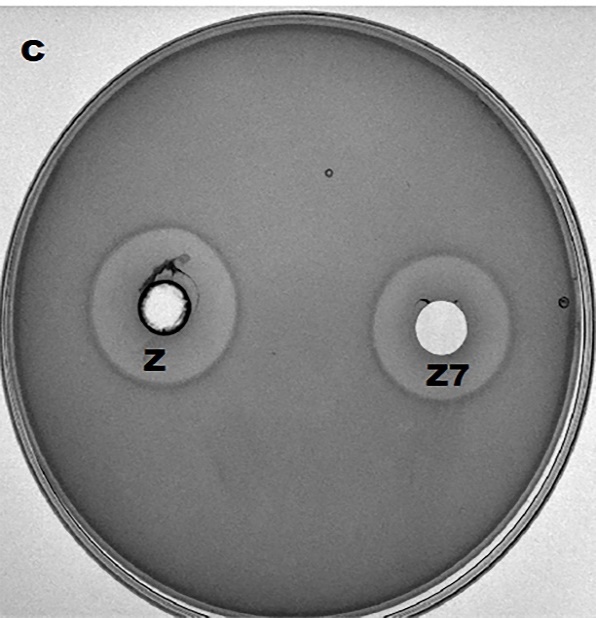


**Figure S1**: **Inhibition of supernatant of *Lactobacillus plantarum* Zhang-LL on *Bacillus subtilis* WB800N, *E.coli* K99, and *Listeria monocytogenes* ATCC54003.** Z, supernatant of *L. plantarum* ATCC54003; Z7, neutralized supernatant of *L. plantarum* ATCC54003. A, *B. subtilis* WB800N; B, *E.coli* K99; C, *L. monocytogenes* ATCC54003.


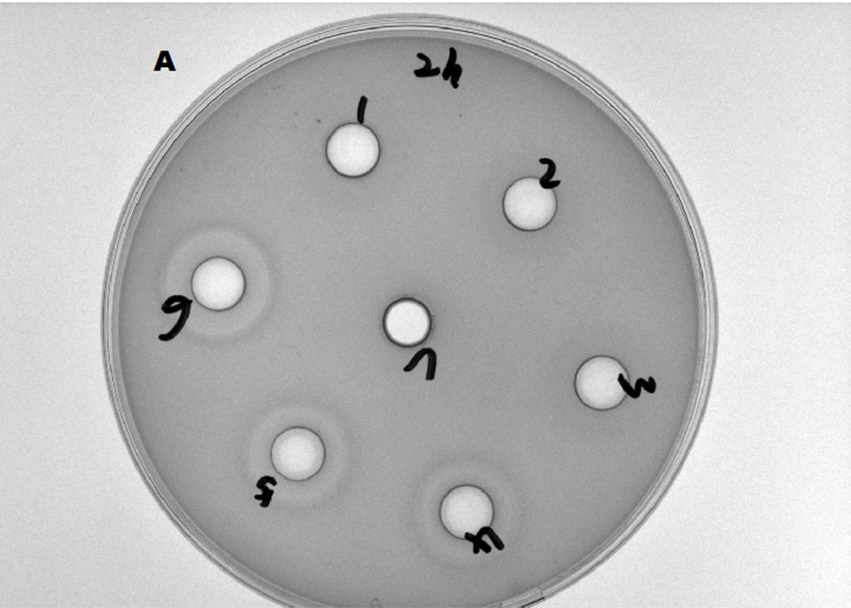


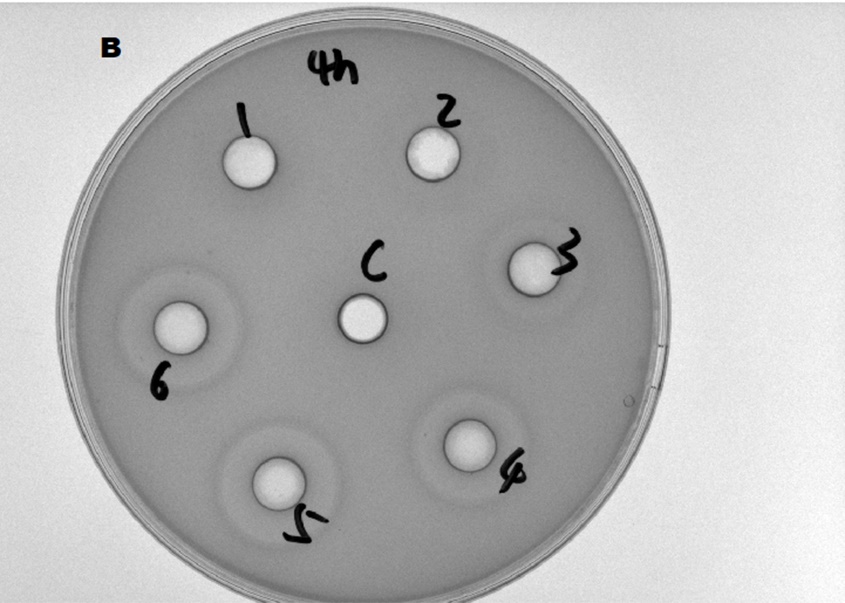


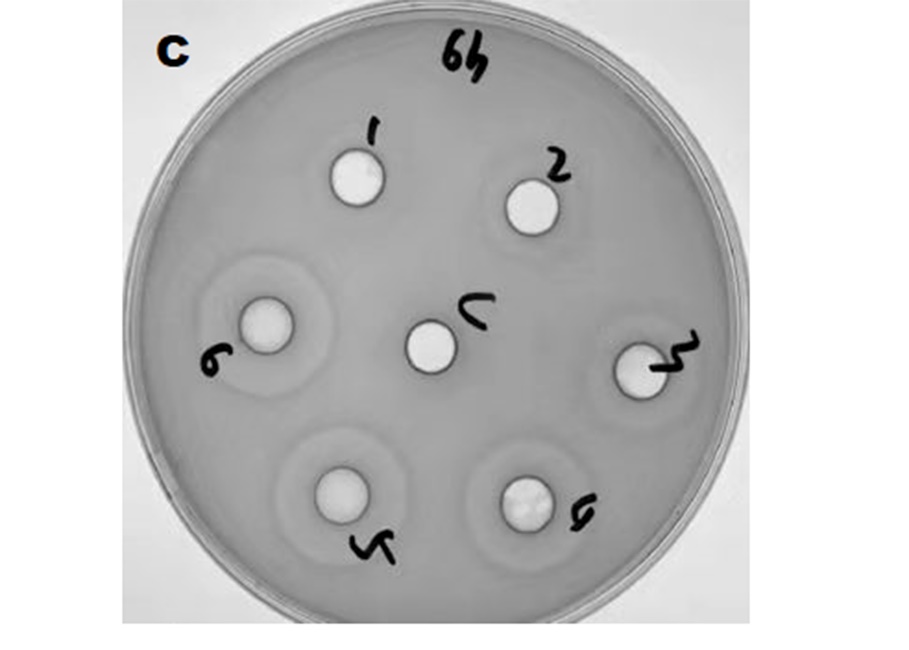


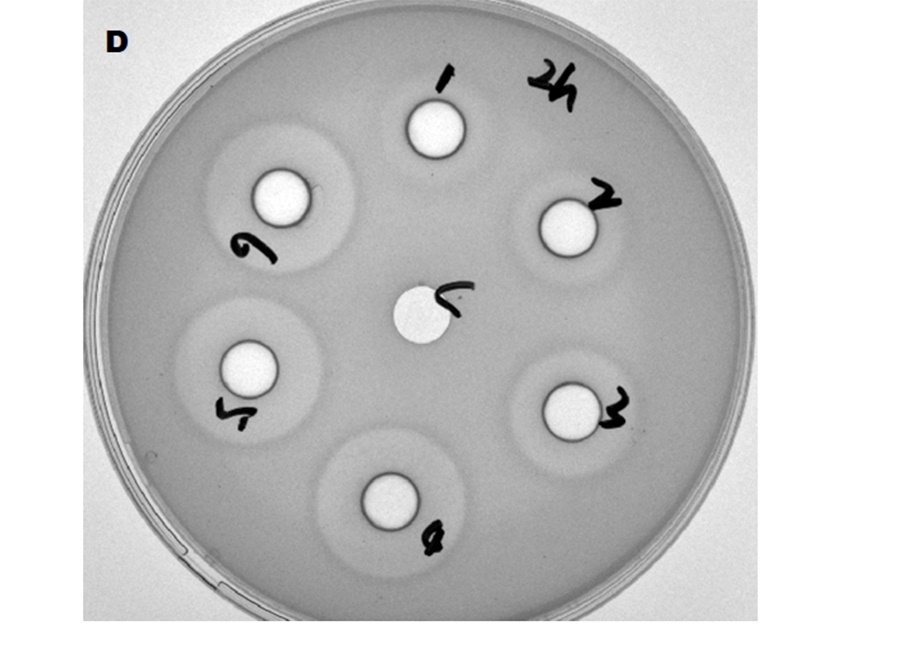


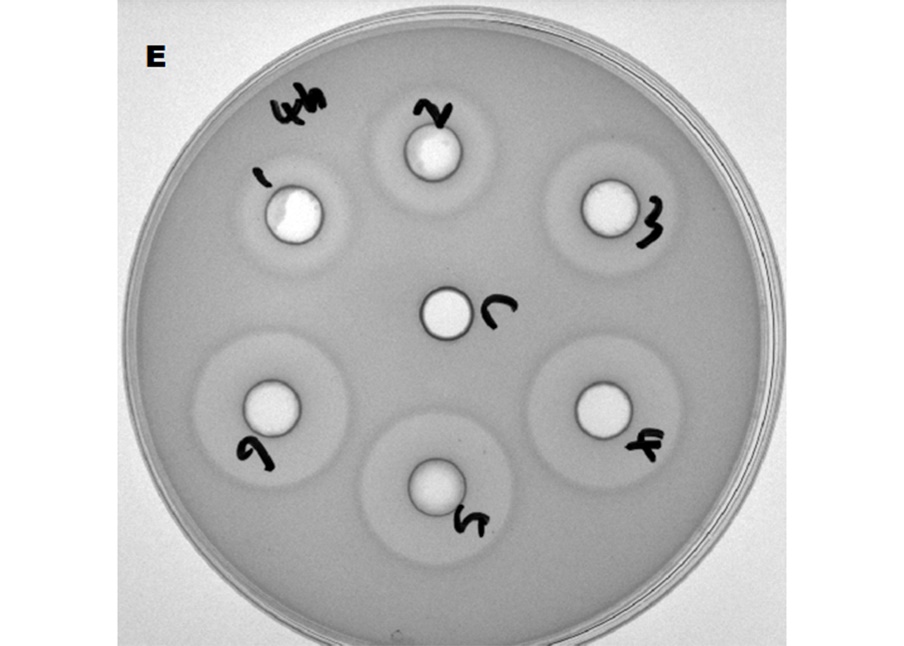

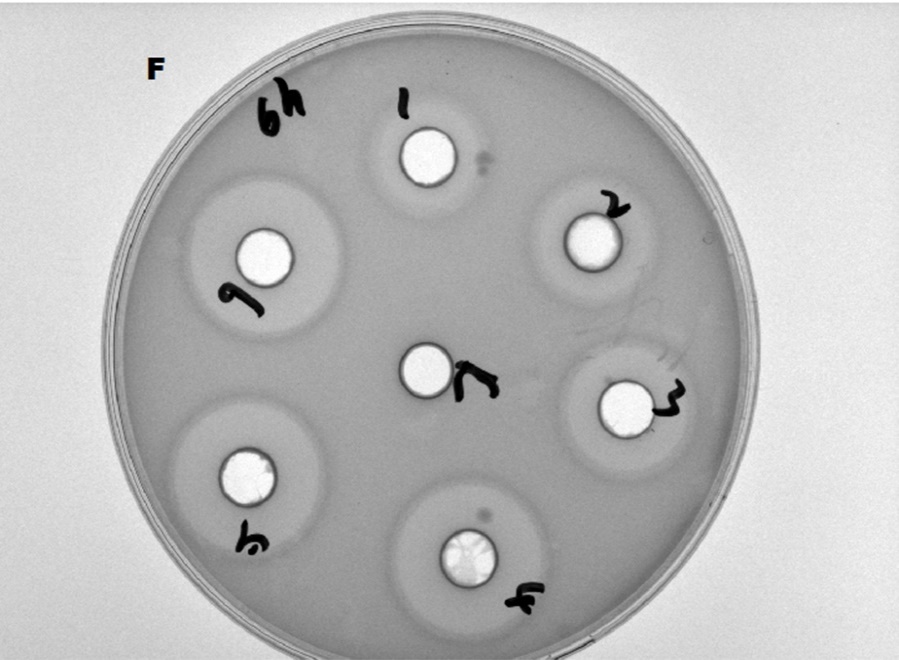


**Figure S2**: **Inhibition of supernatant of various concentration of IPTG and induction time on *L. monocytogenes* ATCC54003.**

*B. subtilis* WB800N/pHT43-*papA1* (*B. subtilis* DBN-SKL-PA1) and *B. subtilis* WB800N/pHT43-*papA2* (*B. subtilis* DBN-SKL-PA2) were induced with IPTG respectively. Samples were collected at 2 h, 4 h, and 6 h. supernatant was subjected to gel diffusion assay. 1, 0.005 mM IPTG; 2, 0.01 IPTG; 3, 0.02 mM IPTG; 4, 0.05 mM IPTG; 5, 0.1 mM IPTG; 6, 0.2 mM IPTG; C, supernatant of *B. subtilis* WB800N/pHT43 inducing with 0.1 mM IPTG for 4 h. A-C, supernatant of *B. subtilis* DBN-SKL-PA1; D-F, supernatant of *B. subtilis* DBN-SKL-PA1. A and D, 2 h; B and E, 4 h; C and F, 6 h.


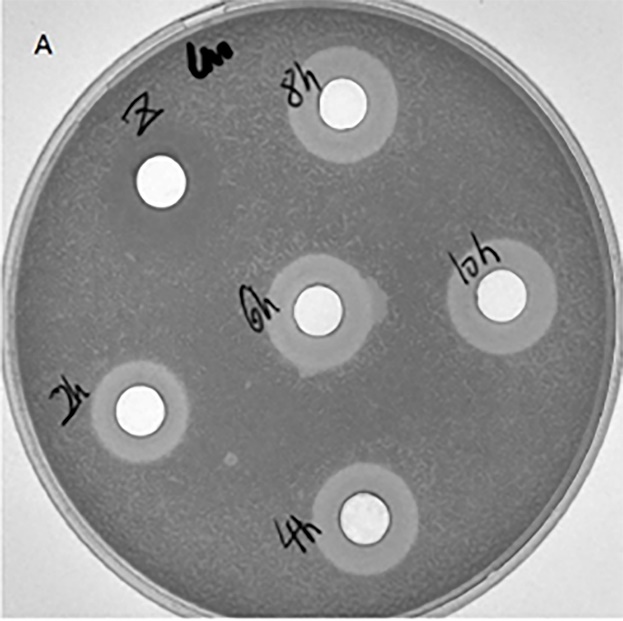

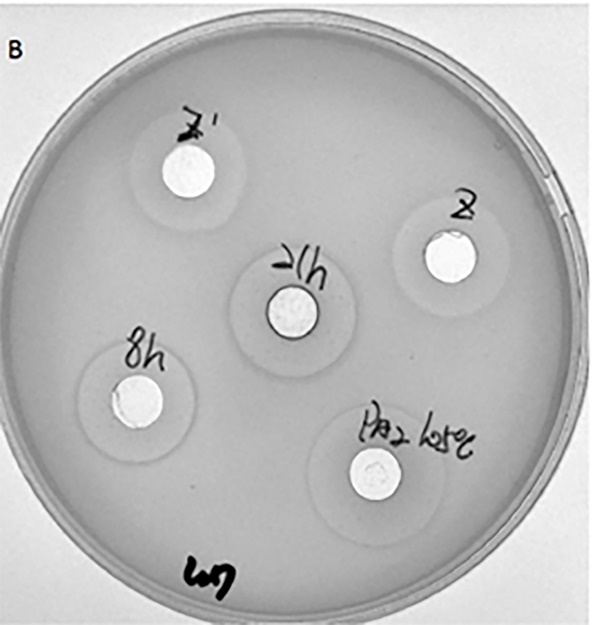


**Figure S3**: **Gel diffusion assay of batch fermentation supernatant on *L. monocytogenes* ATCC54003.** Sample of *B. subtilis* DBN-SKL-PA2 was withdrawn and supernatants were subjected to inhibition assay. A: supernatant of 2 h, 4 h, 6 h, 8 h, and 10 h. B: 8 h, supernatant of fermentation at 8 h; 21 h, supernatant of fermentation at 21 h; Z, supernatant of *L. plantarum* Zhang-LL; Z´, neutralized supernatant of *L. plantarum* Zhang-LL; PA2 105℃, rehydration of 24 h supernatant dry powder.


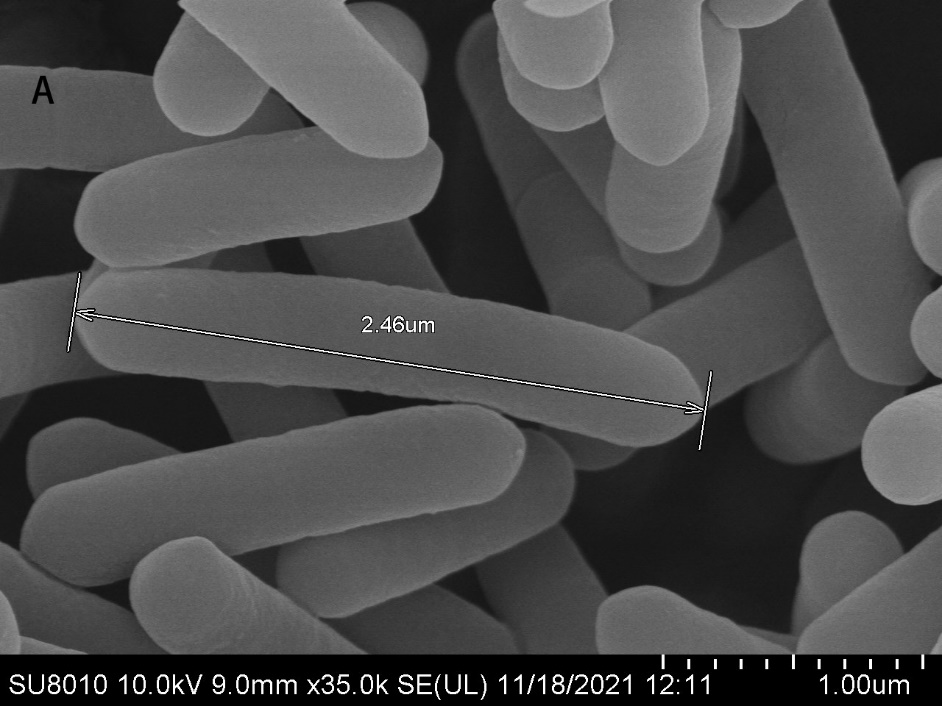


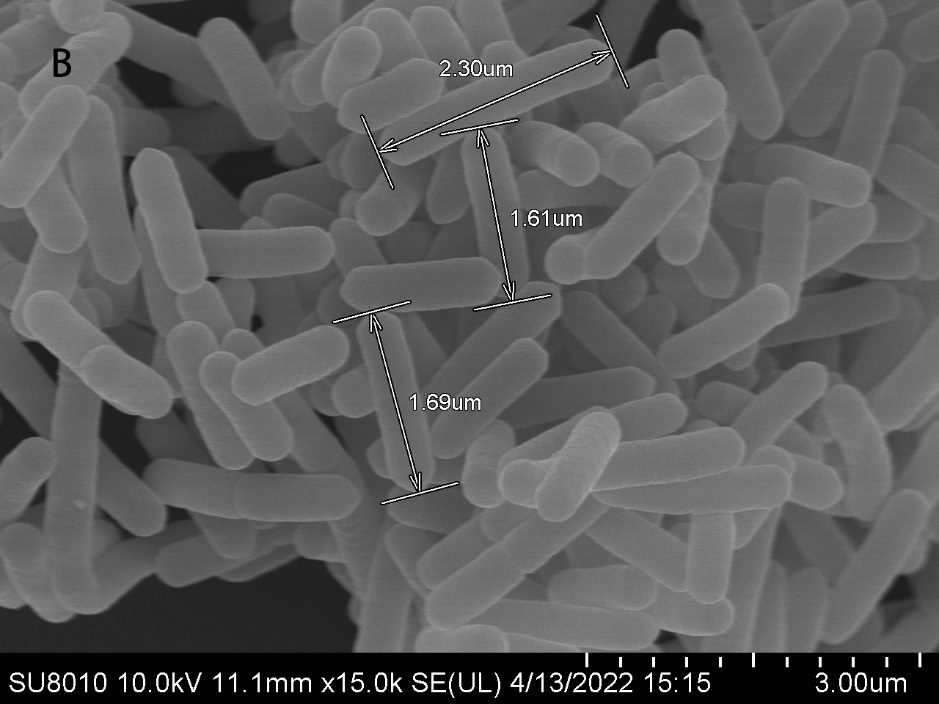


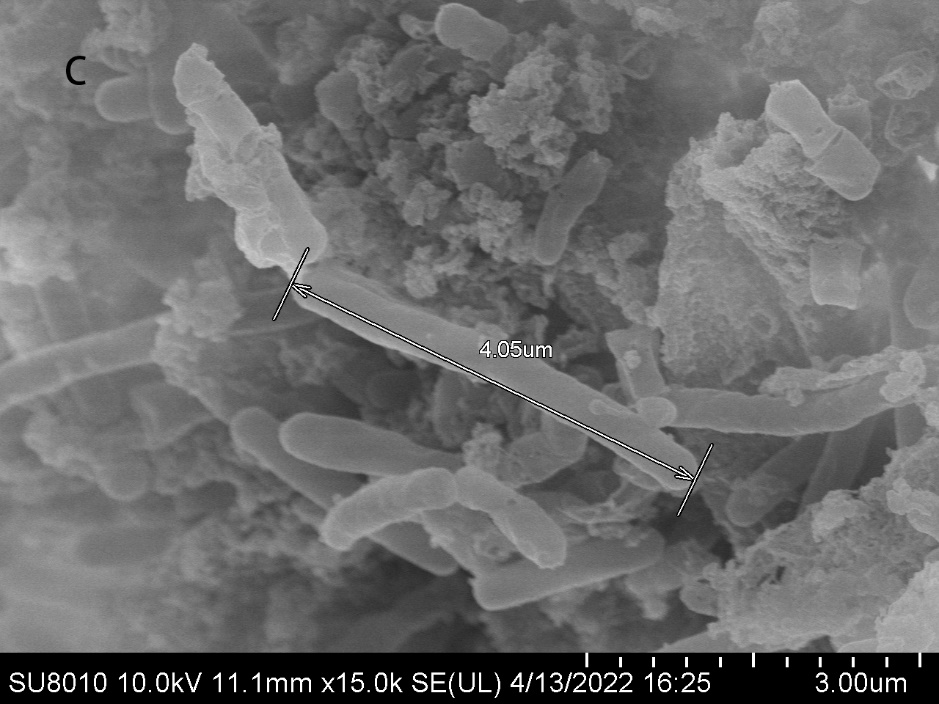


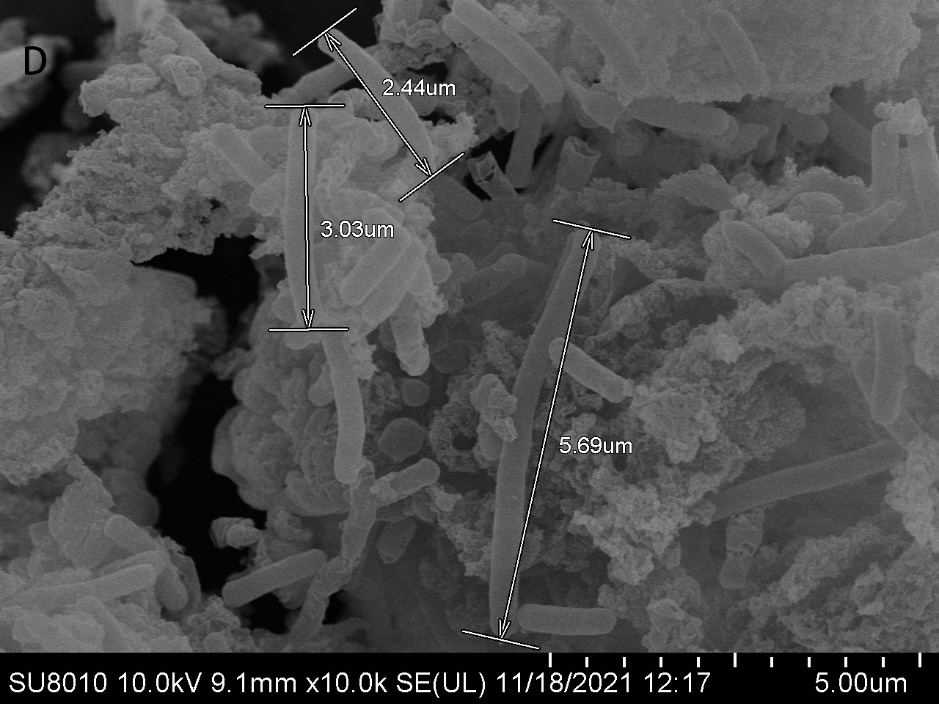


**Figure S4**. **Scan electron microscope of *L. monocytogenes* ATCC54003.** A and B, *L. monocytogenes* ATCC54003 cells; C and D, *L. monocytogenes* ATCC54003 cells incubated with supernatant of recombination protein PA2.
